# Acupoint thread embedding compared to or in combination with biofeedback for treating anismus: A retrospective cohort study

**DOI:** 10.1097/MD.0000000000046143

**Published:** 2025-11-28

**Authors:** Hong Zhi Geng, Xi Ye Mao, Yue Ma, Ke Wu, Bo Chen

**Affiliations:** a Department of Anorectal Surgery, Hepu People’s Hospital, Guangxi Zhuang Autonomous Region, Beihai City, China; b Department of Acupuncture and Moxibustion, Guilin Hospital of Integrated Traditional Chinese and Western Medicine, Guangxi Zhuang Autonomous Region, Guilin City, China; c Department of Anorectal Surgery, Guilin Hospital of Integrated Traditional Chinese and Western Medicine, Guangxi Zhuang Autonomous Region, Guilin City, China.

**Keywords:** acupoint thread embedding, anismus, biofeedback therapy, pelvic floor dysfunction, traditional Chinese medicine syndrome score

## Abstract

Biofeedback therapy (BFT) is the first-line treatment for anismus, a constipation-type defecation disorder characterized by pelvic floor muscle dyscoordination during defecation; however, BFT has limited efficacy and is associated with high recurrence. Acupoint thread embedding (ATE) is a traditional Chinese medicine therapy offering sustained stimulation. We aimed to evaluate the short-term efficacy of ATE, BFT, and their combination in treating anismus, and explore the prognostic value of the traditional Chinese medicine syndrome (TCMS) score. This retrospective cohort study included 150 patients with anismus treated at Guilin Hospital of Integrated Traditional Chinese and Western Medicine between January 2019 and December 2024. Patients were divided into ATE (n = 45), BFT (n = 45), and ATE + BFT (n = 60) groups. The primary outcomes were the Cleveland Clinic constipation (CCC) and TCMS scores at 3-month posttreatment. Secondary outcomes included the patient assessment of constipation quality of life (PAC-QOL) score, total effectiveness rate, and safety, evaluated 3-month posttreatment and at the 3- and 6-month follow-ups. Multivariable linear regression was used to identify prognostic factors. Three-month posttreatment, the ATE + BFT group reported significantly lower CCC, TCMS, and PAC-QOL scores compared with the BFT group and significantly lower PAC-QOL scores compared with the ATE group (*P *< .01). At the 3-month follow-up, the CCC, TCMS, and PAC-QOL scores in the ATE + BFT group were significantly lower than those in the BFT group (*P *< .05); the TCMS and PAC-QOL scores were significantly lower than those in the ATE group (*P *< .01). At the 6-month follow-up, all scores had returned to the baseline levels. The total effectiveness rate was the highest in the ATE + BFT group (85.00%), significantly surpassing that in the BFT group (68.89%, *P *= .03). A ≥5-point reduction in the TCMS score at 3-month posttreatment independently predicted a favorable outcome (*P *< .01). This study demonstrated that the combination of ATE and BFT is significantly superior to either monotherapy alone in the short-term management of anismus, evidenced by greater reductions in CCC, TCMS, and PAC-QOL scores posttreatment. Furthermore, a ≥5-point reduction in the TCMS score was identified as an independent predictor of a favorable prognosis.

## 1. Introduction

Anismus primarily presents as discoordinated relaxation and contraction of the pelvic floor muscles during defecation; it involves multi-link abnormalities, such as neural regulation, muscle function, rectal motility, and structural adaptive changes.^[1]^ This mechanism leads to obstruction of the fecal evacuation pathway, clinically manifesting as a constipation-type defecation disorder, with symptoms including straining during defecation, a sense of incomplete evacuation, and prolonged defecation time.^[1,2]^ Epidemiological studies have indicated that anismus accounts for 27% to 59% of cases of functional constipation.^[3]^

Biofeedback therapy (BFT), as a first-line noninvasive treatment, guides patients to regain pelvic floor muscle coordination through electromyographic sensors. Its core principle is to train the brain to control the coordinated contraction and relaxation of the pelvic floor muscles.^[4]^ BFT can help patients improve sensitivity to rectal sensation, thereby achieving better control over defecation.^[5]^ However, the efficacy of BFT remains limited, with an effectiveness rate of 60% to 70% and a recurrence rate exceeding 30%.^[6]^

Acupoint thread embedding (ATE) is a traditional Chinese medicine therapy that involves implanting absorbable threads into specific acupoints to provide continuous stimulation for disease treatment.^[7]^ Combining the characteristics of acupuncture and modern medicine, ATE offers advantages such as surgical simplicity and long-lasting efficacy.^[8]^ The combined strategy of ATE and BFT achieves a synergistic effect in treating anismus through dual neuromuscular regulation, with its core lying in real time monitoring and adjustment of pelvic floor muscle activity via biofeedback.^[4]^

A critical gap in current research is the lack of comprehensive assessment of the therapeutic efficacy of ATE, BFT, and their combination, as well as the limited exploration of the predictive value of the traditional Chinese medicine syndrome (TCMS) score.^[9]^ More so, current long-term consolidation treatment protocols are unstandardized and require evidence-based validation.

Therefore, this study aimed to evaluate the short-term efficacy of ATE, BFT, and their combination on TCMS, Cleveland Clinic constipation (CCC),^[10]^ and patient assessment of constipation quality of life (PAC-QOL)^[11]^ scores in patients with anismus. We hypothesized that ATE combined with BFT would result in significantly greater reductions in TCMS scores compared with either monotherapy alone (ATE or BFT). We believe the findings of this study will provide evidence-based support for optimizing clinical pathways.

## 2. Materials and methods

### 2.1. Study design and patients

A retrospective cohort study was conducted on clinical data from patients diagnosed with anismus at the Department of Anorectal Surgery, Guilin Hospital of Integrated Traditional Chinese and Western Medicine, between January 2019 and December 2024. Participants were categorized into 3 treatment groups: ATE group (n = 45): received Baliao acupoint therapy combined with pattern-differentiation-based acupuncture. BFT group (n = 45): underwent BFT. ATE + BFT group (n = 60): received combined ATE and BFT. Groups were matched for age, disease duration, severity, baseline pathological changes, and prior treatments for TCMS to minimize confounding effects. The study adhered to the STROBE guidelines^[12]^ and was approved by the Institutional Ethics Committee (KY 2020-001). All participants provided written informed consent in accordance with the Declaration of Helsinki.^[13]^

### 2.2. Patient inclusion

We included individuals who were diagnosed based on the Rome IV International Diagnostic Criteria for Functional Gastrointestinal Disorders for dysfunctional constipation^[14]^ and the TCMS classification of constipation, in accordance with the consensus of integrated Chinese and Western medicine for treating functional constipation (2017)^[9]^; were aged between 18 and 80 years old; had a minimum interval of 1 week between the completion of previous therapies and the initiation of treatment; experienced difficult defecation and exhibited strain during defecation; and had x-ray defecography revealing paradoxical puborectalis contraction.

### 2.3. Case exclusion criteria

Pregnant women; those with chronic systemic diseases, such as cardiac, pulmonary, hepatic, or renal dysfunctions; those with long-term use of other drugs; ATE contraindicated due to skin trauma in the application area, sacrococcygeal or spinal cord injuries, or sacral abnormalities; and individuals with psychiatric disorders were excluded from our study.

### 2.4. Treatment methods

All study participants underwent standardized routine examinations and were provided with conservative management by at least 2 certified colorectal specialists and 2 accredited BFT practitioners within our institution. Efficacy assessments were conducted independently by blinded physicians not involved in the treatment delivery, utilizing a predefined objective evaluation protocol. Standard treatment included dietary modifications, moderate exercise, psychological adjustments, and the establishment of proper bowel habits.

#### 2.4.1. ATE treatment used Baliao points in conjunction with the selection of acupuncture points based on pattern differentiation

The Baliao acupoint in the human body is located in an area extending inward and downward from the high point of the bilateral iliac skeletal spines towards the high bony prominence, also known as the posterior–superior bilateral iliac spine. The upper acupuncture point is situated at the midpoint between the superior–posterior skeletal spine and the posterior midline. Downward palpation along the posterior midline to the second protrusion, corresponding to the second sacral spinous process, identified the secondary acupuncture point. This point is located in a small bony posterior sacral foramen between the posterior–superior iliac spine and the second vertebra. The upper, secondary, middle, and lower acupuncture points were identified by applying pressure with the middle finger at the secondary acupuncture point, positioning the index and middle fingers at the upper and secondary acupuncture points, and evenly extending the remaining fingers.

The main bilateral Baliao acupuncture points were located on the superior (Shangliao [BL31]), secondary (Ciliao [BL32]), middle (Zhongliao [BL33]), and inferior (Xialiao [BL34]) sides. The corresponding acupuncture points for specific conditions were as follows: Quchi (LI11) was used bilaterally, along with Shangjuxu (ST37) for excessive constipation and exuberant heat; Tianshu (ST25) was used bilaterally in combination with Guanyuan (CV4) for cold symptoms; Qi stagnation was addressed bilaterally using Zhongwan (CV12) and Taichong (LR3); constipation accompanied by Qi deficiency was treated bilaterally using Qihai (CV6) and Zusanli (ST36); Geshu (BL17) and Pishu (BL20) were used bilaterally to alleviate blood deficiencies; Yang deficiency was addressed bilaterally by targeting Shenshu (BL23) and Guanyuan (CV4); and Yin deficiency was treated bilaterally using Zhaohai (KI6) and Sanyinjiao (SP6).

#### 2.4.2. ATE procedure

The ATE sterilization package was prepared, and a No.3-0 collagen thread (specification: No.3-0; product standard number: 20120012; manufacturer: Shandong Boda Medical Products Co., Ltd., Shan County, Heze City, China) was cut into segments 0.5 to 1.2 cm long. An acupuncture needle was inserted through the posterior end of the injection needle, and any excess length extending beyond the injection needle was removed. The resulting core needle served as the foundation for thread embedding. The operator retrieved a portion of the core from the rear of a single-use 7-gauge injection needle, used hemostatic forceps to grasp the cut thread segment, and inserted it into the front end of the hollow tube of the syringe needle. Using the right hand, the operator pierced the skin at the selected acupuncture point with the needle containing the thread, pushed it into the muscle layer, and performed lifting and thrusting reinforcing-reducing techniques. After achieving Qi, the practitioner slowly advanced the needle core with their left hand, inserted a collagen thread segment into the muscle layer of the acupuncture point above the sacral foramen, and withdrew the needle with their right hand. Finally, pressure was applied to the exit site using a sterile cotton swab for 1 to 2 minute.

#### 2.4.3. Treatment course

ATE therapy was administered twice monthly, once every 14 day, resulting in 6 sessions per treatment course.

### 2.5. Biofeedback therapy protocol

A Solar Gastrointestinal Compact Biofeedback instrument (Medical Measurement Systems Ltd., Netherlands) was used. Before treatment, patients were administered an enema, instructed to empty their bladder, assumed a lateral position on their right side with knees bent towards the screen, and had their exposed anus disinfected. The balloon was inflated with air until it reached 300 mm Hg. Specialised manometer system balloons were attached, and the lateral hole was closed after normal percolation from the balloon exit hole. A condom was placed over the balloon, coated with paraffin oil, and slowly inserted into the anus to a depth of approximately 10 cm. The electrodes were connected and applied to the outer side of the upper third of the patient’s left thigh and positioned at the 3 and 9 O’clock positions on both sides of the anus. The BFT scenario trained in Sea World mode was selected, and patients were instructed to follow the commands. This process was repeated 20 times daily. Course of treatment: 1 course every 2 week, followed by a 2-week cessation, for 3 treatment courses.

### 2.6. Observation indicators and efficacy evaluation

Questionnaires were provided to all patients before treatment and within 24 hour after 1 month of treatment, and indicators were recorded for 3 and 6 consecutive months posttreatment to summarize and evaluate the results. TCMS scores (0–17 points) were assessed using the methodology outlined in the 2017 consensus on integrated TCM treatment for functional constipation.^[9]^ Treatment was deemed effective if significant improvement was observed in the patients’ primary symptoms and signs. Clinical recovery: symptoms and signs fully resolved, efficacy index ≥ 90%; significant effect: symptoms and signs markedly improved, 60% ≤ efficacy index < 90%; effective: symptoms and signs improved, 30% ≤ efficacy index < 60%; ineffective: no improvement, efficacy index < 30%. Total effectiveness rate = significant effect + effective. The efficacy index was calculated using the formula: ([pretreatment score − posttreatment score]/pretreatment score) × 100%.^[9]^ The degree of constipation was determined using the CCC score (0–30 points),^[10]^ while quality of life was assessed using the PAC-QOL score (0–100 points).^[11]^

### 2.7. Statistical methods

SPSS (version 22.0; SPSS Inc., Chicago) was used for statistical analyses. Categorical data are presented as numerical values. Continuous data are expressed as mean ± standard deviation and were assessed for normal distribution using multivariate linear regression analysis. Within-group comparisons were performed using a *t* test, while between-group comparisons were conducted using 1-way repeated-measures analysis of variance with the least significant difference post hoc test. Statistical significance was set at *P* < .05.

## 3. Results

### 3.1. Patient recruitment and baseline characteristics

A total of 150 patients meeting the diagnostic criteria for anismus were enrolled and non-randomly allocated to 3 groups: ATE alone (n = 45), BFT alone (n = 45), and ATE combined with BFT (ATE + BFT; n = 60). Baseline characteristics, including age, sex, symptom duration, CCC score, TCMS score, and PAC-QOL score, were comparable among the 3 groups (all *P* > .05; Table 1).

**Table 1:** Demographic and baseline characteristics of the patients with anismus receiving different treatments.

General information	ATE	BFT	ATE + BFT
Number of cases (mean ± standard deviation)	45	45	60
Age, yr	50.53 ± 13.90	50.31 ± 12.38	50.55 ± 14.81
Sex			
Female	28	29	38
Male	17	16	22
Body mass index, kg/m^2^	22.44 ± 0.51	22.51 ± 0.51	22.69 ± 0.62
Duration of constipation, mo	51.36 ± 29.09	50.91 ± 42.67	50.38 ± 43.55

ATE = acupoint thread embedding, BFT = biofeedback therapy.

### 3.2. Primary outcomes: symptom severity scores

#### 3.2.1. CCC score

Three-month posttreatment, the ATE + BFT group demonstrated the greatest improvement in constipation severity (Δ = –4.92, –48.96%, odds ratio [OR] 0.52, 95% confidence interval [CI]: –2.67 to –0.62; *P* < .01), which was significantly superior to that in both the ATE (–39.44%) and BFT groups (–31.69%). The treatment efficacy waned in all 3 groups during the 3-month follow-up period. Notably, the ATE + BFT group exhibited the most pronounced reduction in the CCC score, which declined from –48.96% to –6.77% (Tables 2–4).

**Table 2:** Symptom severity and quality of life scores in the different treatment groups at various time points.

Outcome measure	Group	Baseline	Treatment			Follow-up	
			First	Second	Third	3 mo	6 mo
CCC scores	ATE	10.04 ± 2.32	8.73 ± 2.38	7.31 ± 2.69	6.08 ± 2.88	9.51 ± 2.53	10.40 ± 2.07
	BFT	9.91 ± 2.29	9.46 ± 2.52	8.60 ± 2.76	6.77 ± 2.84	9.33 ± 2.49	10.33 ± 2.00
	ATE + BFT	10.05 ± 2.18	8.50 ± 2.07	7.02 ± 2.05	5.13 ± 2.22	9.37 ± 2.01	10.45 ± 2.04
TCMS scores	ATE	9.13 ± 2.20	7.49 ± 2.37	7.53 ± 2.39	6.13 ± 2.69	7.89 ± 1.89	10.16 ± 1.84
	BFT	9.84 ± 2.20	8.93 ± 2.29	8.95 ± 2.34	7.27 ± 2.36	9.78 ± 2.01	9.78 ± 2.01
	ATE + BFT	8.92 ± 1.93	7.05 ± 1.94	7.32 ± 2.04	5.40 ± 2.18	6.73 ± 2.03	9.36 ± 2.01
PAC-QOL scores	ATE	74.20 ± 14.41	68.28 ± 14.53	61.51 ± 14.96	54.56 ± 16.34	62.16 ± 15.66	70.33 ± 14.89
	BFT	73.11 ± 14.10	70.80 ± 15.77	65.58 ± 15.95	58.58 ± 16.89	63.44 ± 16.50	71.44 ± 14.57
	ATE + BFT	74.45 ± 13.61	64.35 ± 13.51	56.80 ± 14.92	45.02 ± 14.16	52.70 ± 13.96	68.45 ± 13.54

ATE = acupoint thread embedding, BFT = biofeedback therapy, CCC = Cleveland Clinic constipation, PAC-QOL = patient assessment of constipation quality of life, TCMS = traditional Chinese medicine symptoms.

**Table 3:** Symptom severity and quality of life score changes in the different treatment groups across time points.

Group	Index	Time point	Change amount (Δ)	Percentage change (%)
ATE	CCC	3-mo after treatment	–3.96	–39.44
		3-mo follow-up	–0.53	–5.28
	TCMS	3-mo after treatment	–3	–32.86
		3-mo follow-up	–1.24	–13.58
	PAC-QOL	3-mo after treatment	–19.64	–26.47
		3-mo follow-up	–12.04	–16.23
BFT	CCC	3-mo after treatment	–3.14	–31.69
		3-mo follow-up	–0.58	–6.35
	TCMS	3-mo after treatment	–2.57	–26.12
		3-mo follow-up	–0.06	–0.61
	PAC-QOL	3-mo after treatment	–14.53	–19.87
		3-mo follow-up	–9.67	–13.23
ATE + BFT	CCC	3-mo after treatment	–4.92	–48.96
		3-month follow-up	–0.68	–6.77
	TCMS	3-mo after treatment	–3.52	–39.46
		3-mo follow-up	–2.19	–24.55
	PAC-QOL	3-mo after treatment	–29.43	–39.53
		3-mo follow-up	–21.75	–29.21

Negative percentage values indicate score reductions (improvement), as lower scores for CCC, TCMS, and PAC-QOL correspond to better outcomes.

BFT = biofeedback therapy, ATE = acupoint thread embedding, CCC = Cleveland Clinic constipation, PAC-QOL = patient assessment of constipation quality of life, TCMS = traditional Chinese medicine symptoms.

**Table 4:** Outcome comparison between the treatment groups after treatment and at follow-up.

Outcome measure	ATE + BFT vs ATE OR (95% CI)	*P*	ATE + BFT vs BFT OR (95% CI)	*P*	ATE vs BFT OR (95% CI)	*P*
3-mo after treatment						
CCC score	0.52 (–1.70 to 0.07)	.07	0.52 (–2.67 to –0.62)	<.01	0. 55 (–1.78 to 0.40)	.22
TCMS score	0.47 (–1.67 to 0.20)	.12	0.47 (–2.80 to –0.93)	<.01	0.51 (–2.13 to –0.13)	.03
PAC-QOL score	3.09 (–15.65 to –3.43)	<.01	3.09 (–19.67 to –7.45)	<.01	3.31 (–10.55 to 2.51)	.41
3-mo follow-up						
CCC score	0.50 (–1.70 to 0.29)	.16	0.50 (–2.19 to 0.20)	.02	0.53 (–1.55 to 0.57)	.37
TCMS score	0.38 (–1.94 to –0.45)	<.01	0.38 (–1.92 to –0.43)	<.01	0.40 (–0.78 to 0.82)	.96
PAC-QOL score	3.09 (–15.41 to –3.50)	<.01	3.01 (–16.70 to –4.80)	<.01	3.22 (–7.65 to 5.07)	.69
6-mo follow-up						
CCC score	0.46 (–1.05 to 0.76)	.75	0.46 (–0.87 to 0.94)	.94	0.50 (–0.79 to 1.14)	.71
TCMS score	0.47 (–1.42 to 0.42	.29	0.39 (–1.18 to –0.35)	.29	0.41 (–0.85 to 1.12)	.79
PAC-QOL score	2.81 (–7.44 to 3.68)	.50	2.81 (–8.55 to –2.56)	.29	3.00 (–7.05 to 4.83)	.71

ATE = acupoint thread embedding, BFT = biofeedback therapy, CCC = Cleveland Clinic constipation, CI = confidence interval, OR = odds ratio, PAC-QOL = patient assessment of constipation quality of life, TCMS = traditional Chinese medicine symptoms.

#### 3.2.2. TCMS score

Three-month posttreatment, the ATE + BFT group demonstrated the most significant synergistic improvement in TCM symptoms (Δ = –3.52, –39.46%, OR 0.47, 95% CI: –2.80 to –0.93; *P *< .01), surpassing both the ATE (–32.86%) and BFT groups (–26.12%). At the 3-month follow-up, the ATE + BFT group demonstrated sustained treatment efficacy (Δ = –2.19,–24.55%, OR 0.38, 95% CI: –1.92 to −0.43; *P* < .01) and was the only group showing a significant reduction in the TCMS score during the follow-up. The treatment efficacy in the ATE group partially persisted (–13.58%), whereas that in the BFT group demonstrated almost complete reversal (–0.61%), suggesting that BFT fails to sustain improvements in TCMS scores (Tables 2–4).

Multivariable linear regression analysis revealed that a reduction in the TCMS score of ≥5 points (5/17 points, 29.41% reduction; 90% of cases) at 3-month posttreatment was a significant independent predictor of a favorable prognosis (OR 2.37, 95% CI: 1.52–3.70; *P* = .001; Tables 2–4).

### 3.3. Secondary outcomes: PAC-QOL, total efficacy rate, and safety

#### 3.3.1. PAC-QOL score

Three-month posttreatment, the ATE + BFT group exhibited the greatest reduction in the PAC-QOL score (Δ = –29.43, –39.53%, OR 3.09, 95% CI: –19.67 to –7.45; *P* < .01) compared with the ATE (–26.47%) and BFT groups (–19.87%). At the 3-month follow-up, the ATE + BFT group maintained the most notable improvement in PAC-QOL scores (–29.21%, OR 3.01, 95% CI: –16.70 to –4.80; *P *< .01) compared with the ATE (–16.23%) and BFT groups (–13.23%; Tables 2–4).

#### 3.3.2. Summary of trends

**Figure 1.**
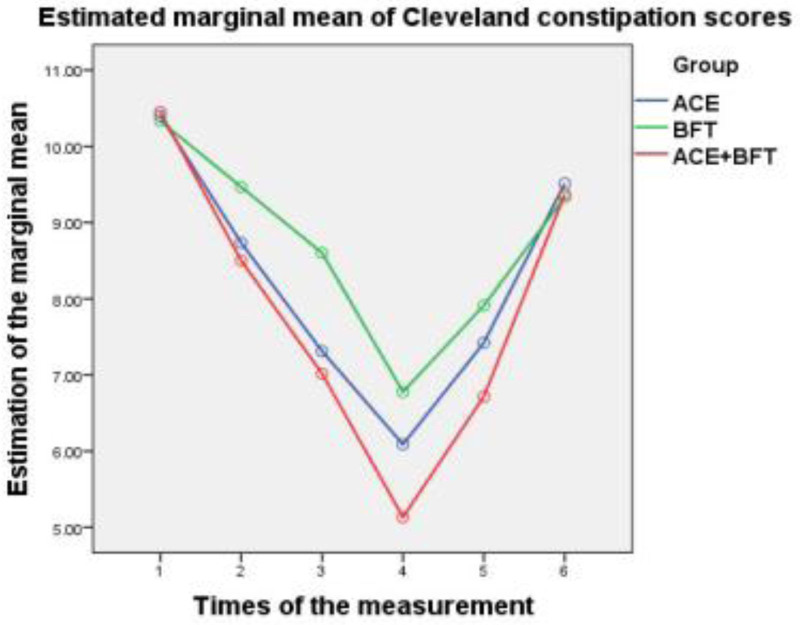
Trend charts of CCC scores in the ATE, BFT, and ATE + BFT groups. Relative to baseline, after 1, 2, and 3 mo of treatment and at the 3- and 6-mo follow-up evaluations, the therapeutic effects show a group trend: ATE + BFT > ATE > BFT. After 3 mo of treatment, the CCC scores decrease to the lowest point; at the 3-mo follow-up, they begin to increase; and at the 6-mo follow-up, they increase to the baseline level. The results show that the therapeutic effects of ATE, BFT, and ATE + BFT are sustained for over 3 mo. ATE = acupoint thread embedding, BFT = biofeedback therapy, CCC = Cleveland Clinic constipation, PAC-QOL = patient assessment of constipation quality of life, TCMS = traditional Chinese medicine symptoms.

**Figure 2.**
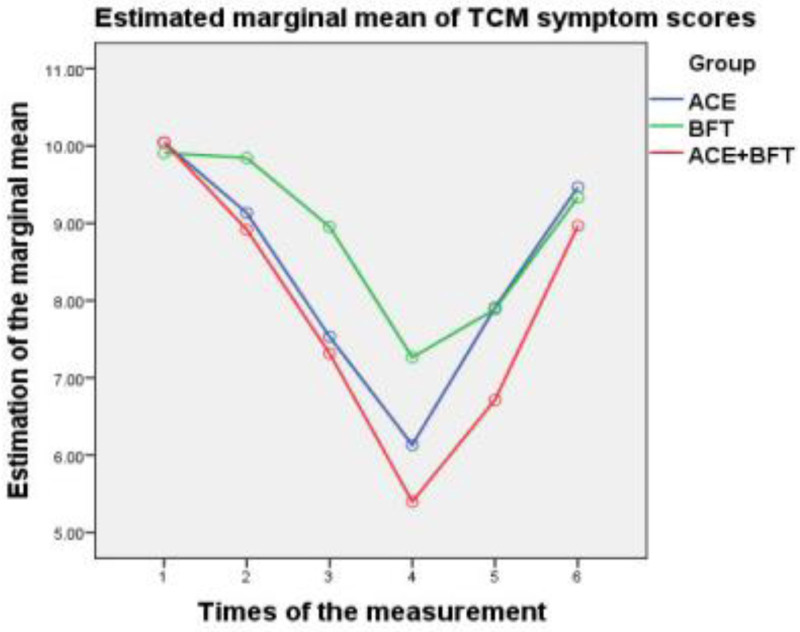
Trend charts of TCM scores in the ATE, BFT, and ATE + BFT groups. Relative to baseline, after 1, 2, and 3 mo of treatment and at the 3- and 6-mo follow-up evaluations, the therapeutic effects show a group trend: ATE + BFT > ATE > BFT. After 3 mo of treatment, the TCMS scores decrease to the lowest point; at the 3-mo follow-up, they begin to increase; and at the 6-mo follow-up, they increase to the baseline level. The results show that the therapeutic effects of ATE, BFT, and ATE + BFT are sustained for over 3 mo. ATE = acupoint thread embedding, BFT = biofeedback therapy, CCC = Cleveland Clinic constipation, PAC-QOL = patient assessment of constipation quality of life, TCMS = traditional Chinese medicine symptoms.

**Figure 3.**
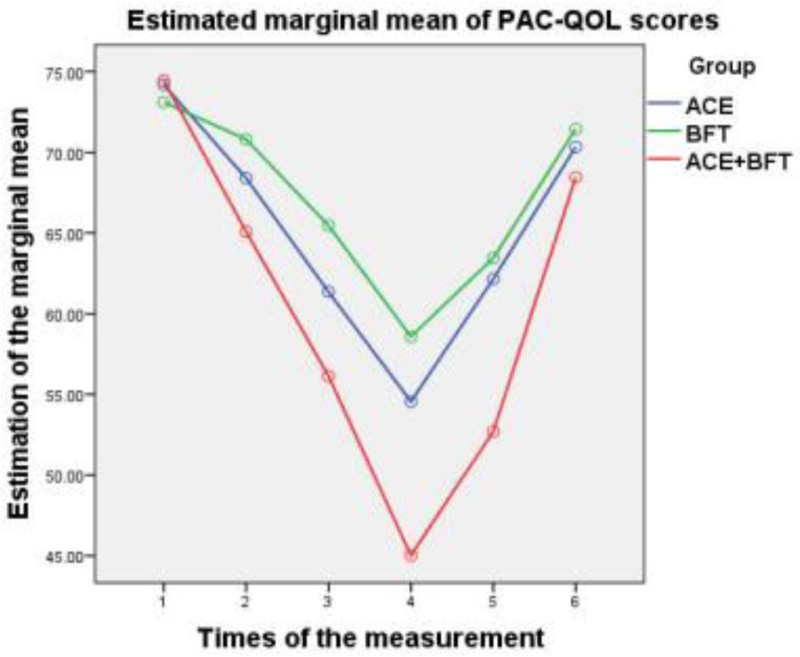
Trend charts of PAC-QOL scores in the ATE, BFT, and ATE + BFT groups. Relative to baseline, after 1, 2, and 3 mo of treatment and at the 3- and 6-mo follow-up evaluations, the therapeutic effects show a group trend: ATE + BFT > ATE > BFT. After 3 mo of treatment, the PAC-QOL scores decrease to the lowest point; at the 3-mo follow-up, they begin to increase; and at the 6-mo follow-up, they increase to the baseline level. The results show that the therapeutic effects of ATE, BFT, and ATE + BFT are sustained for over 3 mo. ATE = acupoint thread embedding, BFT = biofeedback therapy, CCC = Cleveland Clinic constipation, PAC-QOL = patient assessment of constipation quality of life, TCMS = traditional Chinese medicine symptoms.

Regarding the posttreatment efficacy, the ATE + BFT group consistently showed the largest score reductions (greatest improvement) for all indices, followed by the ATE group, then the BFT group (Figs. 1–3). At the 3-month follow-up, the ATE + BFT group maintained the highest residual reduction, particularly for CCC (Fig. 1), TCMS (Fig. 2), and PAC-QOL (Fig. 3) scores; comparably, the BFT group had the most transient short-term effects, with TCMS scores nearly returning to the baseline values. At the 6-month follow-up, the CCC (Fig. 1), TCMS (Fig. 2), and PAC-QOL (Fig. 3) scores had returned to near-baseline levels in all of the groups, indicating that quality of life improvements was not maintained long-term.

#### 3.3.3. Subgroup analysis

The total effectiveness rates at 3-months posttreatment were 75.56%, 68.89%, and 85.00% for the ATE, BFT, and ATE + BFT groups, respectively. The therapeutic effects of ATE + BFT were sustained up to the 3-month follow-up based on the TCMS scores (Figs. 1–3). One-way repeated-measures analysis of variance revealed that the total effectiveness rate in the ATE + BFT group was significantly higher than that in the BFT group (*P* = .03; Table 5).

**Table 5:** Three-month posttreatment efficacy index in the different treatment groups.

Groups	Number of cases	Clinical efficacy index (cases [%])			
		Significant effect	Effective	Ineffective	Total effective
ATE	45	5 (11.11)	29 (64.44)	11 (24.44)	34 (75.56)
BFT	45	2 (4.44)	29 (64.44)	14 (31.11)	31 (68.89)
ATE + BFT	60	11 (18.33)	34 (56.67)	9 (15.00)	45 (85.00)

ATE = acupoint thread embedding, BFT = biofeedback therapy.

#### 3.3.4. Safety

Nine mild adverse events were reported during the treatment period: 3 cases of hematoma and thread discharge at the embedding site (ATE group); 2 cases of abdominal pain during treatment sessions (BFT group); and 4 composite events (ATE + BFT group). No serious adverse events occurred. The incidence of adverse events did not significantly differ among the groups (*P* = .32).

## 4. Discussion

This study demonstrated the advantages of combining ATE and BFT for the treatment of anismus, supported by 3 key findings that provide novel evidence-based insights. The combination of ATE and BFT achieved a significantly greater reduction in constipation severity – measured by CCC, TCMS, and PAC-QOL scales – compared with BFT alone. This robustly supports our hypothesis that combining neuromodulatory approaches offers superior symptom control over single BFT interventions. The efficacy of the combined approach indicates a synergistic effect between these Eastern and Western treatment modalities in alleviating the core symptoms of anismus. Crucially, a ≥5-point reduction in the TCMS score emerged as a significant prognostic indicator, establishing the TCMS scale as a validated prognostic tool in anismus for the first time and providing a quantitative indicator for stratifying treatment. Furthermore, the combination therapy demonstrated sustained efficacy in specific domains. While all groups exhibited a return of CCC, TCMS, and PAC-QOL scores towards the baseline levels at the 3-month follow-up, the TCMS scores in the ATE + BFT group were reduced to a significantly lesser extent, with markedly less rebound. This suggests that the combination therapy provides more stable long-term benefits in ameliorating TCMS manifestations.

The observed synergy between ATE and BFT likely stems from their complementary actions on distinct pathophysiological pathways underlying anismus. Embedding biodegradable threads at specific acupoints (e.g., BL31–BL34) provides continuous physical stimulation, analogous to the “De Qi” effect in acupuncture.^[15]^ This stimulation modulates meridian function and improves local Qi and blood circulation, potentially activating immune responses.^[16]^ Critically, proximity of the BL31–BL34 acupoints to the sacral nerve roots (S2–S4) enables targeted neuromodulation.^[17]^ Thread stimulation likely influences the defecation reflex arc via cytokine signaling and mechanotransduction, enhancing parasympathetic tone and counteracting puborectalis dyssynergia – mechanisms analogous to those underlying sacral nerve stimulation for colonic dysfunction.^[18]^ ATE primarily addresses chronic muscle spasm and structural changes, promoting long-term regulation.^[19]^

BFT facilitates real time correction of recto–anal coordination abnormalities (“brain–muscle disconnect”) through electromyographic feedback, directly improving muscle dyssynergia;^[3]^ it primarily targets acute functional coordination deficits to promote short-term learning. The sustained neuromodulatory effects of ATE – potentially involving upregulation of neurotrophic factors, such as brain-derived neurotrophic factor and glial cell line-derived neurotrophic factor^[20]^ – may create a favorable “primed” neural environment that enhances the neuroplasticity induced by BFT. Essentially, ATE targets the structural/tonic component (muscle spasm) while BFT addresses the functional/phasic component (motor coordination), synergistically correcting the structure–function abnormalities characteristic of anismus.

The findings of the present study notably advance the current evidence base: ATE + BFT achieved a markedly higher effectiveness rate (85.0%) than that typically reported for BFT (30–71%),^[5,6]^ highlighting its potential in refractory cases where BFT fails (contrasting with the 64% efficacy).^[4]^ Furthermore, ATE induced significantly greater TCMS score improvement than did BFT, underscoring the specific value of TCM approaches in functional anorectal disorders.^[8]^ In the acute (3 months) period following ATE + BFT, optimal improvements were observed across all 3 outcome measures, with a particularly significant relative reduce in the PAC-QOL score by–39.53%. This finding highlights the synergistic effect of ATE combined with BFT. Notably, the CCC score exhibited the most pronounced attenuation during the follow-up period, indicating that while the combined therapy yields excellent short-term efficacy, attention should be directed toward maintenance strategies (e.g., extending the treatment course or implementing follow-up interventions).

The implementation of ATE + BFT combination therapy holds significant implications for practice and resource allocation: the prognostic value of the TCMS score enables identification of patients most likely to benefit from combination therapy (e.g., a ≥5-point reduction in the TCMS score from baseline, with Qi stagnation/Yin deficiency patterns), facilitating precision medicine. We recommend prioritizing ATE + BFT for patients having treatment failure with 8 weeks of BFT; the significantly lower treatment frequency required for ATE (once every 2 weeks) compared with intensive BFT regimens (e.g., 5 sessions/wk) can substantially reduce outpatient visits. Modeling suggests a potential reduction in resource utilization, making this approach particularly advantageous in resource-constrained settings; and given the observed persistence of improved symptoms beyond 3 months during follow-up (noted in CCC, TMCS, and PAC-QOL scores), we suggest novel maintenance regimen involving a session of ATE + BFT consolidation therapy administered at 8 to 10-week intervals following an initial course of 3 ATE + BFT sessions, replacing the traditional consolidation schedule (performed at 6 weeks and 3, 6, and 12 months).^[21]^

### 4.1. Limitations and future directions

While these findings are promising, several limitations merit consideration: the retrospective nature of the study introduces potential selection bias, although propensity score matching was employed to mitigate this; the lack of high-resolution anorectal manometry and surface electromyography data limited our ability to quantify the precise neuromuscular coordination changes underlying the clinical improvements; and the partial rebound in CCC, TMCS, and PAC-QOL scores at 6 months underscores the challenge of sustained efficacy and necessitates exploration of optimized maintenance strategies (e.g., maintenance ATE protocols). Future research should prioritize: prospective randomized controlled trials incorporating high-resolution anorectal manometry and quantitative electromyography to objectively measure neuroplastic changes and muscular coordination; defining the optimal ATE treatment interval based on collagen thread degradation kinetics and sustained effect duration modeling; and evaluating less invasive neuromodulation techniques (e.g., transcutaneous tibial nerve stimulation and percutaneous posterior tibial nerve stimulation) to balance efficacy, cost, and patient adherence.^[22]^

## 5. Conclusions

This study provides compelling evidence supporting an integrative model combining Eastern neuromodulation with Western biofeedback, ATE + BFT, offering a viable foundation strategy for interdisciplinary management of anismus. The key advantage of the combined treatment lies in the fundamental synergy derived from the complementary mechanisms of action: ATE provides sustained neuromodulation targeting chronic muscle spasm and neural tone via principles of TMCS, while BFT facilitates active motor relearning and functional coordination through principles of Western neurophysiology. This integration of whole-system regulation with long-acting stimulation (TCMS) and precise, targeted, active retraining achieves optimized efficacy and safety profiles, enhances patient experience, and improves long-term outcomes. The ATE + BFT regimen presents a valuable treatment strategy for anismus and offers a promising paradigm for the integrative management of pelvic floor dysfunction.

## Acknowledgments

We gratefully acknowledge the advice of Prof Jianghong Dai for the statistical analysis at the College of Public Health, Xinjiang Medical University.

## Author contributions

**Data curation:** Yue Ma.

**Formal analysis:** Yue Ma.

**Investigation:** Bo Chen.

**Methodology:** Bo Chen.

**Resources:** Ke Wu.

**Writing – original draft:** Hong Zhi Geng.

**Writing – review & editing:** Xi Ye Mao.
